# Supplementary material for: Oxamate Attenuates Glycolysis and ER Stress in Silicotic Mice
Source: Int J Mol Sci. 2022 Mar 10;23(6):3013. doi: 10.3390/ijms23063013 (PMC8953611; doi:10.3390/ijms23063013)
Supplement: Supplementary file 1 [file ijms-23-03013-s001.zip › ijms-1602744-supplementary.pdf]

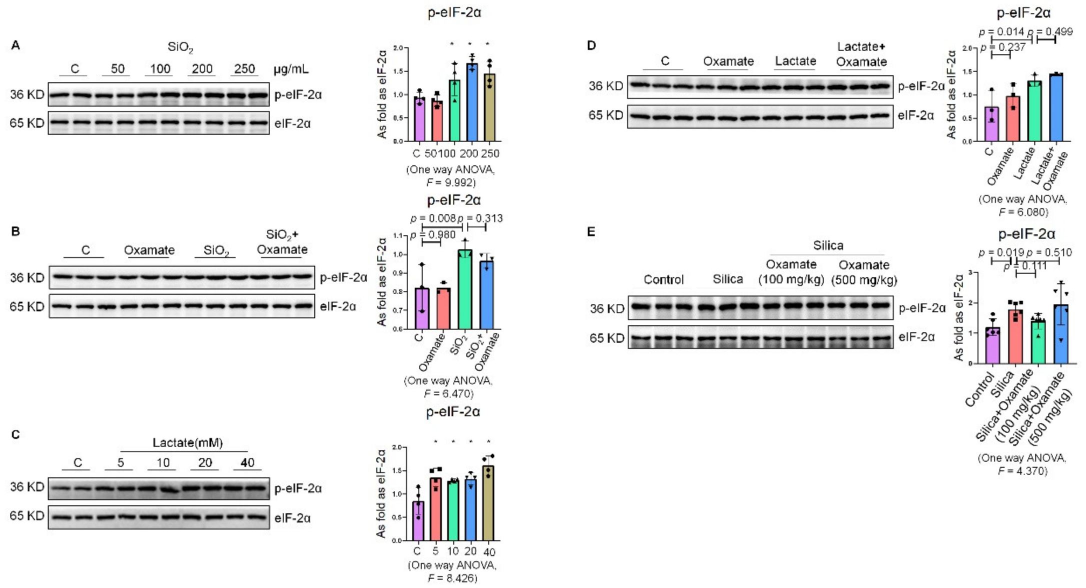

Figure S1. (**A**) Expression levels of p-eIF-2α in NR8383 cells treated with SiO_2 at different doses measured by Western blotting. *Compared with control group, $p < 0.05$. Data are presented as the mean ± SD, n = 4 per group. (**B**) Protein expression of p-eIF-2α in NR8383 cells treated with oxamate, SiO_2, and SiO_2 plus oxamate measured by Western blotting. Data are presented as the mean ± SD, n = 3 per group. (**C**) Expression levels of p-eIF-2α in NR8383 cells treated with lactate at different doses measured by Western blotting. *Compared with control group, $p < 0.05$. Data are presented as the mean ± SD, n = 4 per group. (**D**) Protein expression of p-eIF-2α in NR8383 cells treated with oxamate, lactate, and lactate plus oxamate measured by Western blotting. Data are presented as the mean ± SD, n = 3 per group. (**E**) Expression levels of p-eIF-2α in mice lungs measured by Western blotting. Data are presented as the mean ± SD, n = 6 per group.
